# Temporal dynamics in microbial soil communities at anthrax carcass sites

**DOI:** 10.1186/s12866-017-1111-6

**Published:** 2017-09-26

**Authors:** Karoline Valseth, Camilla L. Nesbø, W. Ryan Easterday, Wendy C. Turner, Jaran S. Olsen, Nils Chr. Stenseth, Thomas H. A. Haverkamp

**Affiliations:** 1 Department of Biosciences, Centre for Ecological and Evolutionary Synthesis (CEES), University of Oslo, The Kristine Bonnevie Building, UiO, campus Blindern, Blindern, Oslo Norway; 2 Norwegian Defence Research Establishment, Kjeller, Norway; 3 Department of Biological Sciences, University of Alberta, Edmonton, Alberta Canada; 4 Department of Biological Sciences, University at Albany, State University of New York, Albany, New York USA

**Keywords:** *Bacillus anthracis*, Metabolism, Metagenomics, Semi-arid, Shotgun sequencing, Taphonomy, Time-series analysis, Sporulation, Microbial diversity

## Abstract

**Background:**

Anthrax is a globally distributed disease affecting primarily herbivorous mammals. It is caused by the soil-dwelling and spore-forming bacterium *Bacillus anthracis*. The dormant *B. anthracis* spores become vegetative after ingestion by grazing mammals. After killing the host, *B. anthracis* cells return to the soil where they sporulate, completing the lifecycle of the bacterium. Here we present the first study describing temporal microbial soil community changes in Etosha National Park, Namibia, after decomposition of two plains zebra (*Equus quagga*) anthrax carcasses. To circumvent state-associated-challenges (i.e. vegetative cells/spores) we monitored *B. anthracis* throughout the period using cultivation, qPCR and shotgun metagenomic sequencing.

**Results:**

The combined results suggest that abundance estimation of spore-forming bacteria in their natural habitat by DNA-based approaches alone is insufficient due to poor recovery of DNA from spores. However, our combined approached allowed us to follow *B. anthracis* population dynamics (vegetative cells and spores) in the soil, along with closely related organisms from the *B. cereus* group, despite their high sequence similarity. Vegetative *B. anthracis* abundance peaked early in the time-series and then dropped when cells either sporulated or died. The time-series revealed that after carcass deposition, the typical semi-arid soil community (e.g. *Frankiales* and *Rhizobiales* species) becomes temporarily dominated by the orders *Bacillales* and *Pseudomonadales,* known to contain plant growth-promoting species.

**Conclusion:**

Our work indicates that complementing DNA based approaches with cultivation may give a more complete picture of the ecology of spore forming pathogens. Furthermore, the results suggests that the increased vegetation biomass production found at carcass sites is due to both added nutrients and the proliferation of microbial taxa that can be beneficial for plant growth. Thus, future *B. anthracis* transmission events at carcass sites may be indirectly facilitated by the recruitment of plant-beneficial bacteria.

**Electronic supplementary material:**

The online version of this article (10.1186/s12866-017-1111-6) contains supplementary material, which is available to authorized users.

## Background

The microbial composition of arid soils across the globe is distinct from other soil environments [1, 2]. The forces that shape arid soil microbial community composition include low water availability, temperature and UV radiation [3–8]. Especially, water restriction has a large influence since it affects several environmental factors, such as salinity, pH, and the availability of (in-) organic matter, which further modulate soil microbial diversity and activity [4, 5, 9–11]. The combination of the above factors may explain why soils in arid environments, such as deserts and arid savannahs, are taxonomically distinct from other soil types [1].

Most research on arid soil microbial diversity is directed to understand the community dynamics under changing environmental conditions, e.g. precipitation changes. However, soil communities can also be affected by the deposition of animal carcasses and their decomposition. This may have a profound impact on the soil microbiome as the carcass influences both biotic (adding new microbes) and abiotic (adding nutrients and moisture) factors. Moreover, after the introduction of a carcass, a succession within the microbial community will occur, changing abundances in accordance with nutrients being released during carcass decomposition [12]. For instance, bacteria belonging to *Proteobacteria* and *Acidobacteria* are the most common in soils during the initial stages of carcass decomposition, while *Firmicutes* are more prominent during active decomposition [13, 14]. The described succession is however, found under experimentally controlled conditions, where carcasses were secured at one location for the entire experiment. In contrast, in natural ecosystems carcasses are often consumed and/or dragged away from the site of death by scavengers [15]. Thus microbes and nutrients may only transiently enter the soil at the site of death [16], which might induce different microbial soil dynamics at natural carcass sites.

The present study investigates the effects of animal carcasses on soil microbial communities after an animal has died of an anthrax infection. The disease anthrax is caused by *Bacillus anthracis,* a gram-positive, rod shaped, sporulating bacterium [17]. This species belongs together with *Bacillus cereus, Bacillus thuringiensis* and several other *Bacillus spp*., to the *B. cereus* group [18], which is commonly found in soil as vegetative cells or as spores [19]. The *B. cereus* group bacteria are indistinguishable from each other using 16S rRNA gene sequences and show high genetic identity (> 99.6%) for certain housekeeping genes [18, 20, 21]. However, *B. anthracis* can genetically be distinguished from the other ‘species’ based on single nucleotide polymorphisms (SNPs) (e.g. in the *plcR* gene [22, 23]) and in most cases by the presence of two virulence plasmids specific to *B. anthracis* pXO1 and pXO2 [24–26]. The lifecycle of *B. anthracis* is different from other *B. cereus* group bacteria in that it predominantly targets, infects and kills mammalian herbivores, instead of insects as found for *B. thuringiensis*. Grazing by herbivores seems to be the main route for transmission in natural settings such as found in the semi-arid savannah in Etosha National Park (ENP), Namibia [27]. After causing the death of its host, *B. anthracis* returns to the soil where it sporulates. Hence, *B. anthracis* spores will often be found in high densities in the top layer of soil where haemorrhagic fluids have leaked from an anthrax carcass [27–29].

Long term measurements at carcass sites in the ENP have identified several processes occurring in and above the soil. It was found that *B. anthracis* cell counts in rhizosphere soils increased in the second year after carcass deposition, but not in surface soils [27]. Those results contrast experimental work where no multiplication of cells was observed in the second year [30]. Furthermore, it was observed that grass biomass and quality increased at the localised area of anthrax carcass sites [27]. This increase in localised plant growth is mostly likely due to nutrient release and/or rhizosphere plant-microbe interactions. The increase in above ground plant biomass results in attraction of grazers, which increases the potential exposure to the pathogen through ingestion of grasses and potentially roots / soil at carcass sites [16, 27]. It is thought that the bacterium sporulates shortly after entry into the soil and that it will stay dormant until favourable conditions arrive [31]. This suggests that there is only a short period, after the entry of *B. anthracis* into the soil, where the pathogen could interact with other microbes or plants to enhance its transmission [16, 30–33]. It is unclear if such interactions influence further transmission of the pathogen. In particular there is a lack of understanding of the dynamics of the (arid) soil microbial community after the influx of animal fluids (e.g. blood, gut contents) with high densities of *B. anthracis* vegetative cells. In order to address how interactions between microbes / plants and *B. anthracis* benefits pathogen transmission, it is important to understand the dynamics of the arid soil microbial community after carcass deposition and influx of *B. anthracis* vegetative cells into the soil.

There are several technical challenges in following both the *B. anthracis* and microbial community dynamics in soil. Currently, DNA-based methods such as 16S rRNA amplicon sequencing and shotgun metagenomics are established methods for the study of complete microbial communities [34]. As mentioned above *B. anthracis* is indistinguishable from other *B. cereus* group species on 16S rRNA gene sequences. Therefore, 16S rRNA amplicons are not suitable for studies aiming to distinguish members from this group. *B. anthracis* and many other microbes can be detected with shotgun metagenomics [35]. Again, *B. anthracis* is genetically highly similar to *B. cereus* group bacteria, which makes bioinformatic identification of these species challenging [18]. Therefore methods able to detect and distinguish various *B. cereus* group species are needed to complement and confirm the shotgun metagenomic results. Such methods include cultivation using selective media (e.g. PLET) and specific qPCR assays [23, 36]. The combination of these methods can then be used to monitor environmental *B. anthracis* populations and to generate hypotheses about possible species interactions, which subsequently can be tested with specifically designed experiments.

Here we present an analysis of soil microbial community composition following leaching of fluids into the soil from two spatially and temporally proximate zebra anthrax carcasses in ENP. The ENP is a semi-arid savannah environment, which for much of the year has sparse water availability, meagre vegetation and limited nutrient resources [37, 38]. Hence, carcass nutrients will likely have great influence on the localised soil microbial community [39]. Large herds of ungulates ensure that every year there is an abundance of carcasses in ENP, often through predation or diseases such as anthrax. It is estimated that up to 400 plains zebras (*Equus quagga*) per year die from anthrax infections in the ENP [40]. Due to the unpredictability of the presence of disease cases, and our interest in controlling for temporal and spatial variability in soil microbiota among study sites, our study was limited to two carcass sites situated proximately in space and time. Our aim was to describe the microbial community succession taking place in the soil after the influx of haemorrhagic fluids containing *B. anthracis* vegetative cells. Shotgun metagenomic sequencing of samples obtained through the first month of decomposition was employed to investigate temporal dynamics of the community structure and function. We investigate the temporal change of the taxonomic composition and the metabolic potential by identifying major metabolic pathways. Finally, we use a combination of techniques (qPCR, cultivation and metagenomic sequencing) to circumvent state (vegetative cell vs. spore) associated challenges to accurately track *B. anthracis* abundances.

## Results

### Carcass information and rainfall recording

On 03.03.2014 we identified two plains zebra (*Equus quagga*) carcasses less than 1 km apart in ENP. Hereafter referred to as Carcass 1 (Ca1) and Carcass 2 (Ca2). Ca1 was intact when sampled on day 0, while Ca2 was minimally scavenged (some intestine was dragged out the anus by vultures). The time of collection (≈ 14:00), the state of both carcasses and only the presence of a few avian scavengers and not mammalian, indicates that both animals were likely dead for less than 12 h and certainly fewer than 24 h. Our study contrasts with other carcass decomposition experiments, since scavenging was not restricted [12–14, 39, 41]. As such, the carcass nutrients and fluids will only leak onto the soil for a short time before scavengers consume soft tissue and move the remains off the site.

After three days both carcasses were completely consumed by scavengers and the bones were found approximately 5 m away from each sampling site. The sampling sites were visited at days: 0, 3, 7, 14, 21 and 30, to collect material for cultivation and DNA extraction. At day zero an uncontaminated control sample (Ctrl0) was taken at both sites to function as a reference sample. The soil in the study area has a alkaline pH around 8.7 ± 0.4 (Additional file 1: Table S1), low moisture and dominance of bacteria (Additional file 2: Figure S1), which is characteristic of arid soils [42].

There was some rainfall at Okaukuejo in the days prior to day 0 followed by little rain until day 18 when heavy rainfalls occurred (day 18–21, Additional file 3: Figure S2).

### Detection of B. anthracis

DNA extraction efficiency of bacterial gram-positive cells and/or spores can be poor [43]. To control for extraction efficiency we spiked soil samples with 3.4 X 10^6^ *B. anthracis* vaccine strain Sterne 34F2 spores (Onderstepoort Biological Products).

**Fig. 1:**
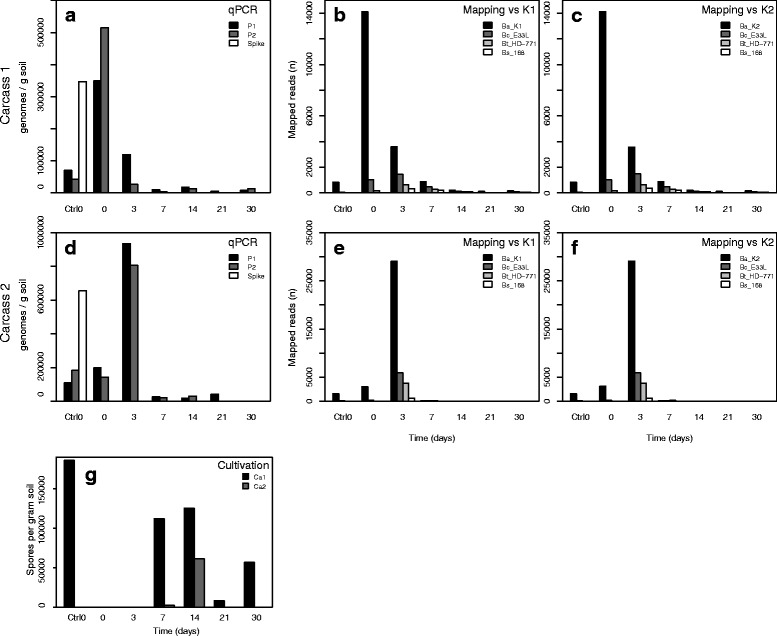
Estimation of *B. anthracis* abundance in soil samples using qPCR, metagenomic reads mapping and cultivation. For all panels, sampling time-points are on the x-axis. **a** and **d** qPCR results with estimated number of *B. anthracis* genomes per gram soil along the y-axis at different time-points (x-axis) for Carcass 1 (Ca1) (**a**) and Carcass 2 (Ca2) (**d**). The spiked sample is control soil from day 0 with 3.4 × 10^6^ Stern34F2 *B. anthracis* spores added. P1 and P2 represent the two replicates at each time-point. **b**, **c**, **e** and **f** shows the results from mapping the metagenomic reads against the *B. anthracis* isolate genomes (K1 /K2) isolated from Ca1 and Ca2 as well as the other reference strains; *B. cereus E33L*, *B. thuringiensis HD-771* and *B. subtilis 168*. Reads matching the references are on the y-axis. **b** and **e** and (**c** and **f**) shows the mapping of the metagenomes against the K1 and K2 strain, respectively as well as the other reference strains. Ca1 in panes (**B** and **C**) and Ca2 in panes (**E** and **F**). (**G)** *B. anthracis* spore counts of soil samples from Ca1 and Ca2 in spores per gram dry soil. Spore counts were estimated by culturing of heat-shocked soil samples and adjusted to dry weight of soil

The qPCR showed that soil samples spiked with *B. anthracis* spores (see Additional file 4: for details) had an estimated recovery of 347,183 (Fig. 1a) and 654,419 (Fig. 1d) genomes representing 10.2% and 19.2% DNA extraction efficiency, respectively (equation in Additional file 4). QPCR on the carcass samples revealed that Ca1 had high abundance of *B. anthracis* at day 0 (Fig. 1a), while a similar peak occurred at day 3 for Ca2 (Fig. 1d). At both sites we find low *B. anthracis* abundances in the Ctrl0 samples. The presence of *B. anthracis* in these samples could either be due to spill-over of *B. anthracis* cells between contaminated and uncontaminated soils (see methods), or *B. anthracis* spores were present in those samples before carcass deposition. With our data it is not possible to determine which explanation is correct.

In order to distinguish *B. anthracis* metagenome reads from those of *B. cereus* and *B. thuringiensis*, mapping of reads was performed using the aln algorithm (Burrows-Wheeler Aligner (BWA-aln)) [44] with very strict mapping parameters (Additional file 4: Methods). In addition, we added closely related *Bacillus spp.* reference strains as bait for sequences not unique to *B. anthracis* [45]. (Note that without the usage of closely related reference strains, mapping of metagenomic reads against *B. anthracis* becomes highly unreliable). Such mapping against the genomes of two *B. anthracis* strains, K1 and K2 isolated from the carcasses studied here [46], resulted in a similar pattern as observed in *B. anthracis* specific qPCR experiments (Fig. 1a, d) with abundances peaking at day 0 for Ca1 and at day 3 for Ca2. For both carcasses there are no significant differences between the mapping results when using the K1 (Fig. 1b, e) or K2 (Fig. 1c, f) genomes. There are few reads (2–94) mapping to *B. cereus*, *B. thuringiensis* or *B. subtilis* in the Ctrl0 samples (Fig. 1, Additional file 5: Table S2). The frequencies for these three species peak at day 3 at both carcass sites, but abundances are lower than for *B. anthracis* (Fig. 1b–f). After day 3 the numbers of reads mapping to all *Bacillus* spp. decrease gradually, except for a slight increase in Ca1 at day 30. A close inspection of the mapping process revealed that many reads with low mapping qualities, e.g. poor alignments due to mismatches, were removed in our final filtering step (Additional file 4: Methods). Removal of reads with mismatches did not change the abundance pattern of our time-series for *B. anthracis*, while there was a significant change for the other species used (Additional file 6: Figure S3). This difference is likely due to the presence of DNA sequences in the metagenomes related, but not identical to the *B. cereus* group genomes available and used as references.

Culturing from soil samples that had been heated to kill vegetative cells, showed no *B. anthracis* spores present at days 0 and 3 (Fig. 1g) in contrast to the qPCR results. Ca1 had >100,000 spores on day 7 and 14, followed by a sharp drop in spores at day 21, and an increase to >50,000 spores at day 30. Ca2 had >2000 spores on day 7 and >60,000 spores on day 14; at the rest of the time-points Ca2 only had <130 spores. Some of these aberrations from the general trend in counts may be products of sampling biases. In addition, the reduction of *B. anthracis* levels on day 21 (Fig. 1g) could also be due to a response to the rainfall prior to day 21 (Additional file 3: Figure S2).

### Temporal changes of GC content and average genome size

Changes in the average % guanine-cytosine (GC) content of metagenomics data indicate changes in microbial community composition [47]. The average GC-content of the time-series metagenomes ranged between 54.6 and 69.0% (Table 1). Interestingly, in both time-series there was a marked drop in average %GC content at day 3 and 7 before it increased again at day 14. This drop was more prevalent in Ca1 than Ca2. This change is due to addition of an extra %GC content peak (≈43%) in the %GC profile, which reduces the average GC-content, and the skewness of the GC-content (Table 1).

**Table 1:** Overview metagenome shotgun samples

Dataset	Time	Raw PE reads	Cleaned PE reads	Cleaned Singleton reads	Average GC-content (%)	GC-content skewness	Average Genome size (Mb)	Genome Equivalents
Carcass 1	Ctrl0^a^	18,736,078	17,119,526	138,893	69.0	−1.52	4.7	1679
	0	18,545,478	18,157,853	189,906	67.7	−1.34	4.2	1975
	3	18,270,652	17,901,288	155,300	60.7	−0.24	3.3	2487
	7	19,794,059	19,333,716	131,415	54.6	−0.08	3.1	2819
	14	18,686,371	18,157,423	227,754	66.4	−0.97	4.0	2034
	21	18,513,371	17,999,309	141,642	67.6	−1.32	4.2	1935
	30	17,526,182	16,999,968	116,608	66.4	−0.96	3.4	2237
Carcass 2	Ctrl0	20,731,918	19,940,019	180,050	68.8	−1.51	4.9	1871
	0	22,832,502	22,263,495	251,377	68.8	−1.67	4.7	2116
	3	20,693,407	20,053,092	167,471	65.2	−0.63	3.8	2386
	7	17,689,524	17,356,265	119,172	64.8	−0.85	4.1	1912
	14	18,078,980	17,079,305	168,140	67.5	−1.46	4.1	1894
	21	17,591,012	17,104,126	148,834	68.7	−1.44	4.8	1615
	30	16,364,557	15,672,082	228,390	68.1	−1.33	4.7	1485

^a^Ctrl0 is the control sample on day 0 without blood

The drop in GC content corresponded with a drop in average genome size (AGS) and an increase in genome equivalents (Table 1) [48]. The AGS drop and the shift in average % GC suggests that the carcass soil community composition is changing due to actively growing microbes responding to the carcass nutrients. In addition, the drop in AGS may indicate that the metabolic capacity of the soils changes over the time-series, since AGS can be used as a measure for the metabolic complexity of an ecosystem [49].

### Taxonomic classification of shotgun sequences

Many tools exist for the taxonomic classification of metagenomic shotgun sequences. Our aim was to use a tool that captures most of the diversity present in the samples. We therefore compared metaxa2 [50], which classifies rRNA sequences in the metagenomes using a modified Silva SSU /LSU database, with metaBIT (wrapper around Metaphlan2) [51, 52], Kraken [53] and MEGAN [54]. Metaphlan2 and Kraken uses a database derived from microbial whole genome sequences, with the difference that Metaphlan2 only uses signature sequences to identify taxa, instead of whole genomes. MEGAN uses the Non-Redundant protein database from NCBI (NR) for classification.

By comparing the relative abundance of reads classified by each tool we found that the tools differed in the amount of reads classified per sample (Additional file 7: Table S3; Additional file 8: Figure S4) [55]. Interestingly, Kraken showed different temporal dynamics with respect to the change in relative abundance of classified reads compared to the other tools. MetaBIT shows an especially large change between days 0 and 3 compared to the other tools (Additional file 8: Figure S4). We assume that in both cases these results are due to the databases used by metaBIT and Kraken. This is also reflected in the number of prokaryotic taxa identified, with MEGAN detecting a maximum of 180 prokaryotic orders, while the three other tools detected fewer taxa (metaBIT:18, Kraken: 121 and metaxa2: 159). This shows that detection of environmental prokaryotes is significantly influenced by database choice.

The results of the above comparison suggest that metaxa2 is the best available choice among the four tools tested here to describe the bacterial and eukaryotic composition of the soil communities in our study (Additional file 7: Table S3), since the database it relies on is both well curated and contains the widest taxonomic range available. Moreover, MEGAN is a good complement to metaxa2, since it uses most reads for taxonomic and functional classification and can provide diversity measures that support community comparisons (e.g. PCoA, clustering etc).

**Fig. 2:**
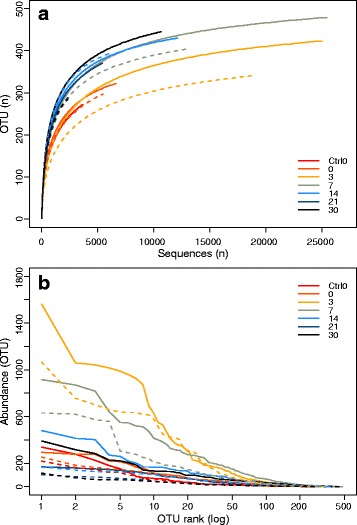
OTU richness of 16S /18S rRNA sequences from soil metagenomic samples. 16S /18S rRNA sequences were extracted with metaxa2 and diversity was analysed with MetaAmp. **a** Rarefaction curves. X-axis indicates number of rRNA sequences, and y-axis shows OTU numbers **b** Rank abundance of OTUs, where OTU rank is on the x-axis and the abundance per OTU is on the y-axis. The solid lines are Ca1 samples and the dotted lines are Ca2 samples. Line colour indicates time-points as indicated in the legend

**Fig. 3:**
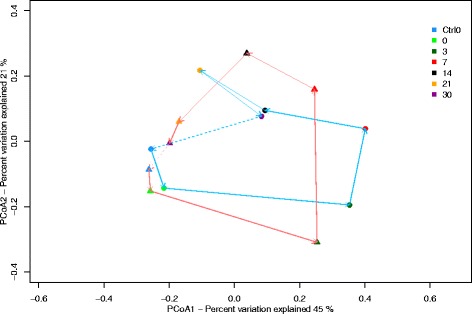
Soil microbial community relationships by time-point and carcass site. Principal coordinate analysis of a distance matrix created by normalised total counts and using Bray-Curtis dissimilarity. Ca1 is represented by the circles and Ca2 by the triangles, the time-points are visualised in different colours and the arrows are pointing in the direction of increasing days, red arrows for Ca1 and light blue for Ca2. The dotted arrows show the relationship between the day 30 to the Ctrl0 sample

The metaxa2 data was used to analyse the community diversity using rarefaction and rank-abundance plots. The number of classified reads per sample varied from 19,661 to 110,996 corresponding to about 50% of the identified rRNA sequences (Additional file 2: Figure S1a). The classified reads were dominated by bacteria, with a relative abundance of 70% ± 0.13% and 69% ± 0.1% for Ca1 and Ca2, respectively (Additional file 2: Figure S1b), which stayed constant regardless of time-point. The rRNA reads clustered into 557 OTUs using 97% similarity cut-off. The two Ctrl0 samples have the lowest number of OTUs, while the highest number of OTUs was found in Ca1 day 7 (Ca1_7) (Fig. 2a). The rarefaction curves (Fig. 2a) indicate that we have captured the dominant taxa present, since the curves start levelling off at around 5400 sequences. Nonetheless, the curves do not become horizontal, which indicates that additional low abundant taxa can still be detected with additional sequencing effort. Ca2 had fewer total rRNA reads than Ca1 at all time-points, except the control samples (Additional file 9: Table S4), which results also in fewer OTUs for Ca2 time-points compared to Ca1. An OTU rank abundance plot (Fig. 2b) indicates that Ca1 has higher OTU abundances for the dominant OTUs at all the time-points compared to Ca2. For both carcasses, the soil microbial community is dominated by a few OTUs on days 3 and 7. At the other time-points the evenness of the community is higher as seen in the day 30 samples where almost all OTUs have similar abundances (Fig. 2b). The OTU abundance distribution for the different time-points suggests different soil communities for each of the samples. And indeed, when we analysed community diversity differences using PCoA, based on MEGAN shotgun read classification, we found a clear separation between the communities (Fig. 3). Here metagenomic sequences from both carcasses follow a similar trend, where they are most divergent from the Ctrl0 and day 0 samples at days 3 and 7, before clustering closer to the Ctrl0 and day 0 sample again on days 14 and 21. Ca1 however, has a deviation from the trend on day 30: this sample is close to the Ca1 day 14 sample. The day 30 sample for Ca2 is similar to both Ctrl0 samples.

### Temporal dynamics of the soil microbial communities

**Fig. 4:**
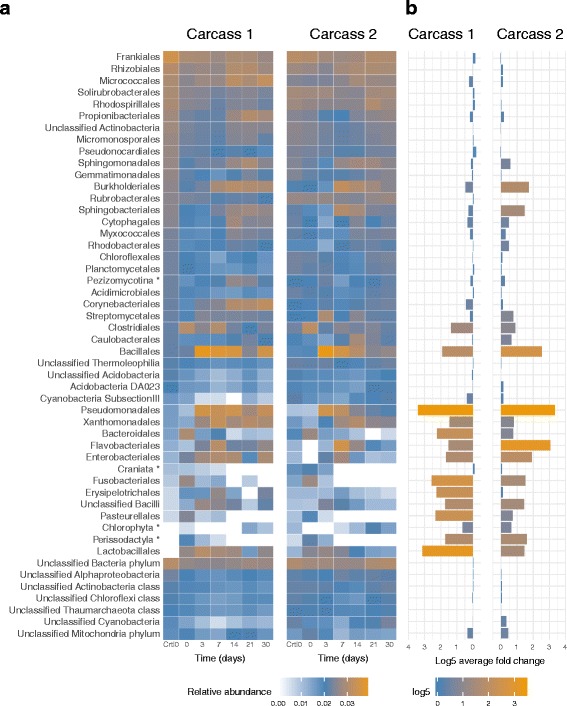
Temporal dynamics of microbial order abundances at carcass 1 and 2. **a** Heatmap visualisation of the relative abundance for the 50 most abundant orders at each time-point for carcass 1 and 2. **b** Barplots that show the log5 average fold change of the raw reads counts across the time-series for the 50 taxa shown in figure (**a**). The taxa in the heatmaps (**a**) are sorted using the order abundances in the control sample of Carcass1 at day 0 (Control). In order to visualise the relative abundances (**a**) values were log5 normalised and then scaled so that the sum of each column equals 1. Eukaryotic orders are marked with *, the remaining orders are bacterial. The bottom seven entries have not been classified to lower phylogeny than Kingdom, Phylum or Class

The 50 most abundant orders from the metaxa2 analysis were extracted and visualised for Ca1 and Ca2 to track fluctuations of the soil community over a 30-day time-period (Fig. 4). For both carcasses there is a clear relative abundance change in part of the microbial community after the influx of body fluids, while another part of the community does not seem to respond to this influx. Relative abundances may reflect an increase or decrease of a taxon due to changes in the abundance of another taxon, which can be misleading. We therefore show in Fig. 4b the log5 average fold changes between the time points of the raw counts for both carcass communities. This illustrates both the average direction of change over time and the average size of the read abundance change.

Several orders show a sharp abundance increase after day 0 (log5 average fold change >1) at both carcass sites. These include *Bacillales* and *Pseudomonodales* species. In contrast, bacteria belonging to orders such as *Frankiales*, *Rhizobiales* and *Solirubrobacteriales* do not show large changes in their read abundances after the blood/nutrient influx at either carcass site. This is despite the large increase in total reads classified (Additional file 2: Figure S1). Finally, there are also orders that show limited increases. The abundance increase can be early (*T* = 0 days) and then decline (e.g. *Clostridiales*, *Bacteriodales*), or increase only later (*T* ≥ 7 days) in the time-series (e.g. *Burkholderiales*, *Corynebacteriales*).

The orders *Bacillales* and *Pseudomonadales* have the highest abundances at days 3 and 7 (Additional file 10: Figure S5), with genera such as *Acinetobacter*, *Lysinibacillus* and *Kurthia* being dominant (Additional file 9: Table S4). These orders are present at all time-points for both carcasses, but are especially abundant on days 3–14. Many of the genomes in the NCBI database from these genera are small (Size <3 Mbp) and their presence can explain the drop in average genome size (AGS) that we observed at day 3 (Table 1).

Between days 14 and 21 the relative rRNA read abundance for *Bacillales* drops at Ca1 and Ca2 by 33 and 23%, respectively. For *Pseudomonadales*, we identify a drop of 17 and 13% for Ca1 and Ca2. In total 9 orders show a relative rRNA read abundance drop (>10%) by day 21 in Ca1 and 12 orders in Ca2. At day 30 the abundances of both *Bacillales* and *Pseudomondales* increased again in Ca1, but not in Ca2. Interestingly, for the “stable” orders there was an overall increase in relative rRNA read abundances at day 21 and a subsequent drop at day 30.

The abundance changes observed at days 21 and 30 are likely influenced by heavy rainfall (16–103 mm) recorded at all the weather stations in the ENP in the three days prior to sampling day 21 (Additional file 3: Figure S2). Thus, we assume that the abundance increase at day 30 for many orders could be a response to this heavy rainfall. Moreover, the precipitation could explain the species abundance profile changes since it can result in short-term changes in the microbial community [56].

In addition to bacterial orders that are normal for soils, we also observed representatives of several bacterial orders likely introduced from the zebra. For instance, *Fusobacteriales* are present in the first days for both Ca1 and Ca2, where they are most abundant on day 0 for both samples. They are completely absent on day 14 and 30 in Ca1 and from day 7 onwards in Ca2. The increase in abundance for this order on day 0 is most likely due to the introduction of *Fusobacterium* spp. such as *Fusobacterium equinum*, which is a known inhabitant of the oral cavity and lower respiratory tract of horses [57]. In Ca1 sequences classified to the order *Pasteurellales* are highly abundant on day 0, but completely disappear after day 7. Their abundance at day 0 is mainly caused by *Actinobacillus* spp. and *Pasteurella caballi* (Additional file 9: Table S4). The later species is a commensal of the upper respiratory tract of horses [58].

### Microbial community metabolism

**Fig. 5:**
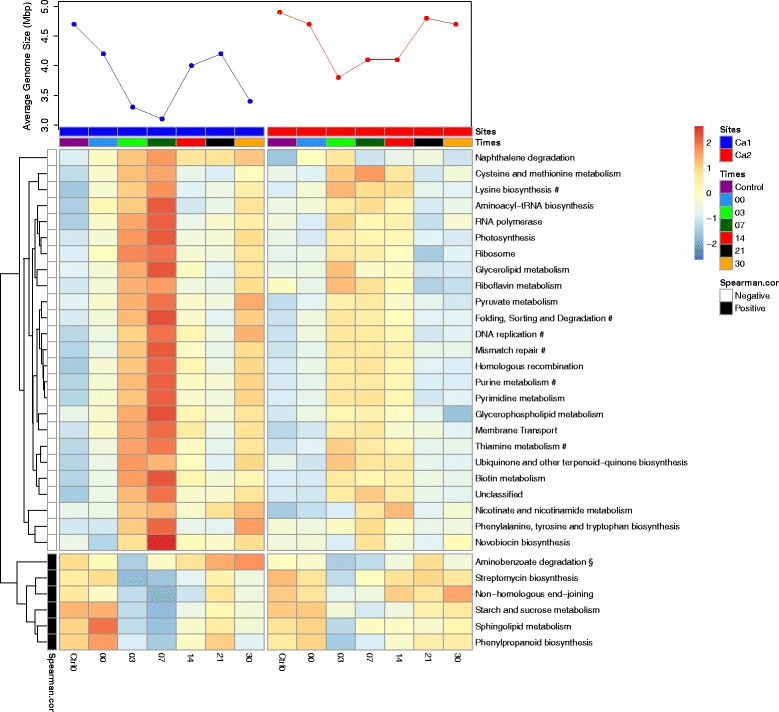
Heatmap of significantly different metabolic pathways per time-point. KEGG metabolic pathways significantly correlating with AGS for microbial communities of Ca1 and Ca2. Pathways were determined by MEGAN classification. KEGG pathways abundances were normalised with DESEQ2 [96] and correlated to the AGS using the Spearman correlation method with the False Discovery Rate (FDR) test to calculate probabilities (FDR cut-off at 0.05). KEGG-pathways correlating positively or negatively with a *p*-value <0.05 are shown for Ca1 (blue) and Ca2 (red) for all the time-points, the AGS is shown in the top panel. # indicates pathways that are significant in both Ca1 and Ca2, § indicates pathways that are significant in Ca2, and the rest are only significant in Ca1. Pathway abundances were centred and scaled per row and positive and negative correlations were clustered based on the sign of the Spearman correlations

The soil metagenomes at the early time-points show a decline in AGS compared to the control sample and the later time-points (Fig. 5). AGS is ecologically informative, as in general, soil bacteria have larger genomes than specialised, opportunistic, parasitic and symbiotic bacteria [59]. Thus AGS change suggests taxonomic and metabolic changes taking place within the microbial community. We therefore correlated KEGG pathway abundances of 112 pathways with AGS to study the temporal change of metabolic potential in the metagenomic time-series. For Ca1 we identified 29 pathways that were significantly correlated with AGS (*p* < 0.05), with 23 being specific to Ca1 (Fig. 5, Additional file 11: Table S5, Additional file 12: Table S6). Six pathways showed a negative correlation with AGS for both Ca1 and Ca2. For the Ca2 metagenomes we identified 7 pathways correlated with AGS, with only one pathway (aminobenzoate degradation) positively correlated with a large average genome size in Ca2 but not Ca1. In Ca1 six pathways show a decrease in abundance that is correlated with the decrease of AGS (Fig. 5, top panel). These pathways show an abundance drop at days 3 and 7 and then a subsequent increase. In Ca2 these pathways show a similar pattern (Additional file 11: Table S5, Additional file 12: Table S6). For the metabolic pathways: novobiocin metabolism (KEGG map00401), streptomycin metabolism (KEGG map00521) and naphthalene degradation (KEGG map00626), the genes identified are not unique for the pathways. For example, no reads were mapped to the genes involved in the final step of novobiocin production and only a few reads to the final step of streptomycin production. The later examples highlight the difficulty in functional annotation of shotgun metagenomic data and indicate the need for cautious interpretation of such results [60, 61].

## Discussion

Shotgun metagenomic sequencing, which allows detection of seemingly all species in a community based on the presence of their DNA, is generally considered a superior method to culturing [1, 62, 63]. This is because it addresses “the great plate count anomaly”, which claims that only about 1% of the microorganisms seen during microscopy can be cultured [64, 65]. However, DNA-based analyses of microbial communities also have technical pitfalls, particularly when studying spore-forming microorganisms such as *B. anthracis,* which can result in large discrepancies between actual and estimated abundances [43]. For *B. anthracis* this is further confounded by the high levels of DNA sequence identity shared with other members of the *B. cereus* group.

The technical pitfalls of DNA-based analyses are evident from our data, where *B. anthracis* spores could readily be cultured from our samples, but were not as easily detected in metagenomes without using a very strict mapping approach. The onset of *B. anthracis* sporulation occurs within the first 72 h after carcass deposition and can continue up to eight days post-mortem [29, 66]. The qPCR data (Fig. 1) indicates that over time *B. anthracis* abundance peaks before almost completely disappearing from the samples. These results, together with the cultivation data, suggest that the reduced quantities of *B. anthracis* observed in the qPCR experiments are due to reduced DNA extraction efficiency (because of sporulation) [67]. This also suggests that abundances of *B. anthracis* and similar organisms are likely underestimated in the metagenomes at several time-points due to low extraction efficiency. Nonetheless, the metagenomes still captured a large part of the microbial community present in the carcass site soils, and can therefore be used to study the temporal changes taking place.

### Why does a carcass promote plant growth?

The data generated reflects the temporal dynamics of the soil microbial community in a natural ecosystem after inoculation of a pathogen (*B. anthracis*), other host-associated microbes and nutrient influx from zebra carcasses. Turner et al. [16] showed that carcass sites in the ENP have higher quality and more abundant vegetation, which could be related to higher levels of phosphate, nitrogen and lower pH than the surrounding soils.

The microbial communities at both carcass sites show a clear response to the influx of bodily fluids, with similar community composition (at order level) of the dominant taxa for the two carcasses with only minor variation in abundances (Fig. 4). Shifts in the community are indicated by several observations: changes in relative abundance of classified reads between the samples (Additional file 3: Figure S2), changes of the AGS of the community studied (Fig. 5) and by variation in OTU diversity and abundance for each of the time-points (Fig. 2). The AGS decreases in the first week of the sample period before increasing again, suggesting a community shift towards copiothrophic bacteria in the first week (Table 1). This is expected as copiothrophic bacteria are better adapted to local increases in nutrient availability than typical soil bacteria and therefore can increase drastically in a short amount of time as observed here [68].

In contrast to the copiothrophic bacteria, the typical soil microbial community consisted of orders like *Frankiales*, *Rhizobiales* and *Solirubrobacterales* (Fig. 4), which remain relatively stable throughout the sample period. This suggests that these bacteria do not need, or are unable to use, the nutrients from the carcass. Moreover, *Rhizobiales* are nitrogen-fixing bacteria living in symbiosis with plants that thrive under nitrogen poor conditions [69]. The extra nitrogen provided by an animal carcass would make them less competitive in soils. *Frankiales* spp. can be plant symbionts, but have also been found to grow on rock beds, which indicate a lifestyle specialized for oligothrophic or extreme conditions [70, 71]. In that respect, it is reasonable that these oligothrophic taxa do not react dramatically to the nutrients of a decomposing animal.

Among the copiotrophic bacteria we see an increase in genera like *Acinetobacter*, *Lysinibacillus* and *Kurthia* in the first week, which is similar to observations in other decomposition studies [12]. These genera belong to the orders *Pseudomonadales* and *Bacillales* and are known to contain species that can be either pathogenic or beneficial for both animals and plants [72–76].

Interestingly, many of the orders reacting quickly to the nutrient influx remain at a relatively high level throughout the sampling period with variations in abundance of genera within orders (Fig. 4). For example, *Xanthomonadales* is abundant at relatively stable levels from day 3 onwards, but the genus *Wohlfahrtiimonas* within this order is only abundant on day 7 at Ca1. *Wohlfahrtiimonas* is a known parasite of different *Wohlfahrtia* spp. (flesh flies) and other such insects. The abundance of *Wohlfahrtiimonas* seen on day 7 for Ca1 may be a result of an increased number of insect hosts appearing through the decomposition process. This example indicates that short-term species composition changes might be due to local conditions in the soil and are not necessarily due to direct competition or sample variation.

Nonetheless, after day 7 other bacterial orders, such as *Burkholderiales,* increase in abundance. This order is typically found in higher abundances in the rhizosphere of plants than in the surrounding soils because they actively feed on plant root exudates [77–79]. Furthermore, *Burkholderiales* are known to have members that are plant growth-promoting bacteria (PGPB), like the *Bacillales* and *Pseudomonadales* genera described above [80–82]. Turner et al. [16] showed that decomposition of carcasses improves soil fertility and enhances plant growth. Our results suggest that this process is promoted by the soil microbial community, which shows a sharp increase in the abundance of orders known to have PGPBs. The promoting functions of PGPBs are many, such as nitrogen fixation, siderophore and phytohormone production, phosphorus solubilisation, and the suppression of plant diseases [79]. *Bacillus anthracis* is an obligate-killer pathogen, able to transmit only by killing its host. Interestingly, by taking advantage of the nutrient- and microbial-driven stimulation of plant growth occurring at carcass sites, transmission of *B. anthracis* is enhanced since this plant growth is an attractive food source for herbivores [16].

### Microbial metabolism in arid environments

To get a functional overview of the microbial communities in our study we investigated how metabolic pathways correlate with changes in AGS over time (Fig. 3). Several of the positively correlating pathways can be linked to environmental stressors typical for arid soils, such as desiccation, high temperatures and (UV) radiation. For instance, starch and sucrose (S&S) metabolism is essential for the synthesis of the disaccharides sucrose and trehalose, which can play a role in prokaryotic desiccation resistance [83]. The importance of DNA repair in the surface soil exposed to high radiation levels is reflected in the positive correlation of the non-homologous end joining (NHEJ) repair system, which is also needed for survival during quiescent states such as in the spore stage [84–86].

The presence of other positively correlating pathways can be explained by their role in nutrient acquisition. Arid soils have low organic matter content and most of it comes from decaying plant material [56]. The positive correlation of the aminobenzoate degradation pathway probably reflects its involvement in the degradation of recalcitrant compounds such as lignin.

Many of the pathways negatively correlated with AGS are essential pathways involved in membrane transport, DNA metabolism (transcription, translation, replication, repair and recombination), amino-acid, lipid, carbohydrate and cofactor metabolism (Additional file 13: Figure S6). This reflects the dominance of opportunistic bacteria, especially at day 3–7, with small genomes that lack the arid soil ‘specialist’ pathways discussed above.

Overall, the analysis of the functional metabolism shows a clear change between the different time-points. The “normal” microbial soil community metabolism in ENP is represented by species that have genes that are involved in dealing with stress encountered in arid soils. The carcass fluids and nutrients activate many dormant or low abundant copiothrophic lineages present in the soil, which is reflected by the pathways needed for proliferation especially present in the first week of our experiment. These two states of the soil community, dormant vs. active, are typical for arid environments where water input stimulates in a pulse-like manner microbial activity and if sufficient even plant growth [56]. Rainfall gives enough moisture to activate both plant growth and the microbial decomposition compartment of the soil, providing plants with necessary nutrients [56, 87, 88]. However, in contrast to rainfall, animal carcasses not only provide moisture to the soil, but also additional nutrients. This pulse of nutrients and moisture allows the microbial community at very local scales to break down carcass nutrients, laying the foundation for enhanced plant growth directly and in the future [16, 56].

## Conclusion

Our work is the first study to use shotgun metagenomics to describe the temporal changes in the soil microbial community after deposition of anthrax carcasses. We observed that the metagenomic approach is not the best way to study *B. anthracis* in a natural setting as it sporulates quickly making detection via DNA extraction difficult. In addition, it is too genetically similar to its close relatives such that most currently available tools to analyse (meta-) genomic sequences are not able to differentiate between such similar organisms. Nevertheless, the metagenomic data allows us to assess how this pathogen influences the microbial soil community.

The metagenomic time-series analysis indicated that carcass introduction stimulated the increase in abundance of part of the microbial soil community. This could play an indirect role in the future transmission of *B. anthracis* by stimulating the growth of taxa known to have PGPBs. The taxonomic shift in the microbial community was accompanied by shifts in the metabolic potential. The typical semi-arid soil community was involved in stress evasion, while the carcass introduction increased pathways involved in proliferation. Nonetheless, the time-series showed that within a month the semi-arid bacterial soil community was similar to the community at *T* = 0 with respect to taxonomic and metabolic composition.

Our work provides a background to study the ecology of spore-forming pathogens in a natural setting using culture-independent methods. It highlights the difficulty of using DNA based approaches to study spore-forming organisms such as *B. anthracis* in arid soils where they are dormant and poorly detectable.

## Methods

### Carcass and sample information

Sample collection in ENP was done after receiving permission from the Ministry of Environment and Tourism of Namibia (permit number: 1857/2013). Two fresh (likely <24 h since death) plains zebra (*Equus quagga*) anthrax carcasses (Ca1 & Ca2) were found on 03.03.2014 between Sprokieswoud and Charl Marais Dam, 835 m apart at coordinates S19.031/E015.548 (Ca1) and S19.037/E015.553 (Ca2), respectively. Information about ENP, carcass sites and sample collection can be found in the Additional file 4. Soil samples were taken from the area of blood-spill at six time-points (T), starting at T = 0 days (03.03.2014), *T* = 3, 7, 14, 21, 30 days. A control sample (Ctrl0) was also taken from the grids at T = 0 days, avoiding areas covered in blood (Additional file 8: Figure S4).

### DNA isolation and purification

A total of 14 soil samples were collected (7 per carcass). DNA was isolated in two replicates from each sample (P1 and P2) resulting in a total of 14 DNA samples per carcass site. DNA was isolated using the FastDNA® spin kit for soil (MP Biomedicals, Santa Ana, California, USA), following the manufacturer’s protocol with adjustments specified in Additional file 4. The DNA samples were filter sterilised using an Ultrafree® Durapore PVDF 0.1 μM spinfilter (Millipore, Darmstadt, Germany).

DNA was shipped dry, following an ethanol precipitation, from ENP to Norway (see Additional file 4: for details). Upon arrival DNA was re-suspended in DES buffer from FastDNA® spin kit for soil. Samples were concentrated and purified using Agencourt® AMPure® XP beads (Beckman Coulter, Beverly, Massachusetts, USA), and treated with a PowerClean® Pro DNA clean-Up Kit, (MO BIO Laboratories, Carlsbad, California, USA) to remove any remaining inhibitors.

### Metagenome sequencing and quality control

Environmental DNA samples were sequenced at the Norwegian Sequencing Centre (NSC) using Illumina MiSeq® (Illumina Inc., San Diego, California, USA). A 250 bp paired-end sequencing library (400 bp insert length) was generated using a Regular TruSeq® adapter ligation kit (Illumina Inc., San Diego, California, USA). Fastq files were processed using cut-adapt (v1.8) [89] and prinseq-lite (v0.20.4) [90] (See Additional file 4: for settings). Metagenomic read GC-content was calculated using infoseq (EMBOSS v. 6.5.7 [91]). The infoseq output was used to calculate average GC-content and GC-content skewness (timeData package version: 3012.100) using R-Studio (v3.3.0).

The average genome size (AGS) of each metagenome was estimated using MicrobeCensus on clean paired-end files (Additional file 4: Methods). The likelihood of sampling a universal single copy is inversely correlated with the AGS of a metagenomes [48, 92]. Thus universal single copy abundance will show significant differences when AGS between metagenomes differ.

### Bacillus anthracis quantification from soils

Two methods were used to quantify *B. anthracis* in the soil samples; serial dilution culturing and quantitative PCR (qPCR) (see Additional file 4: for details).

The genome coverages of the *B. anthracis* isolate K1 and K2 genomes (Accession numbers: LBBZ00000000 and LBCA00000000 [46]) isolated from the Ca1 and Ca2 sites respectively, were investigated by mapping metagenomic sequences using the Burrows-Wheeler Aligner (BWA v0.7.8) [44]. The genomes of *B. cereus* E33L, *B. thuringiensis* HD-771 and *Bacillus subtilis* 168 were also used as references (see Additional file 4: for details).

### Taxonomic and functional classification of metagenomic reads

Taxonomic composition of the metagenomes was determined using metaxa2 (v2.0.1) which extracts SSU rRNA sequences [50], MEGAN (v5.10.15) [54], Kraken (0.10.5-beta) [53] and metaBIT [51]. For the details on the comparison of the taxonomic classifiers see Additional file 4. The metaxa2 taxonomic profiles were visualised with a heatmap using ggplot2 in R-studio (see Additional file 4: for settings). Metaxa2 extracted SSU reads were run through the MetaAmp 1.1 pipeline to obtain operational taxonomical unit (OTU) abundances.

Shotgun sequences from each dataset were compared to the NCBI nr database (accessed on 20.01.2016) using Diamond (v0.7.11) [93] and the output was used for taxonomic and functional classification in MEGAN (v5.10.15) [54]. Temporal species composition dynamics of metagenomic samples was examined by principle coordinate analysis (PCoA) in R-Studio (v3.3.0). Changes in the metabolic potential of the community over time were analysed by correlation of KEGG KO-terms and pathways [94, 95] abundances with AGS size (For details see Additional file 4).

### Data availability

The datasets supporting the conclusions of this article are available in the SRA repository, SRP076706. All sequence data was submitted to Genbank as part of bioproject: PRJNA281298.

## Additional files

Additional file 1: Table S1. Soil nutrients and soil composition at carcass sites. (DOCX 44 kb)

Additional file 2: Figure S1. Abundance and relative abundance of 16S/18S rRNA taxonomically classified into kingdoms. (a) Raw abundance and (b) relative abundance of metaxa2 classified reads. These are the 16S/18S reads extracted and classified by metaxa2. Roughly 50% of the extracted rRNA sequenced did not get assigned to any taxonomy and were assumed to be false positives. (EPS 1341 kb)

Additional file 3: Figure S2. Rainfall at the Okaukuejo field station. The precipitation is measured in mm at Okaukuejo, which is approximately 40 km from the sampling site. Because of the very local rainfalls in the area, this can only be used as an indication of rainfall at our sample sites. The graph shows rainfall from the week before sampling started and to the end of the sampling period, the 2nd of April 2014. The blue arrow indicates the sampling period with sampling days marked in red. (EPS 7 kb)

Additional file 4: A text document contained supplementary methods, equations, results and figure (mentioned only in the supplementary information). (DOCX 896 kb)

Additional file 5: Table S2. Counts of mapped metagenomic reads of each sample against reference genomes. (XLSX 97 kb)

Additional file 6: Figure S3. Estimation of *B. anthracis* abundance in soil samples using qPCR, metagenomic reads mapping and cultivation. For all panels, sampling time-points are on the x-axis. (A and D) qPCR results with estimated number of *B. anthracis* genomes per gram soil along the y-axis at different time-points (x-axis) for Carcass 1 (Ca1) (A) and Carcass 2 (Ca2) (D). The spiked sample is control soil from day 0 with 3.4 × 10^6^ Stern34F2 *B. anthracis* spores added. P1 and P2 represent the two replicates at each time-point. (B, C, E and F) shows the results from mapping the metagenomic reads against the *B. anthracis* isolate genomes (K1/K2) isolated from Ca1 and Ca2 as well as the other reference strains; *B. cereus E33L*, *B. thuringiensis HD-771* and *B. subtilis 168*. Reads with mismatches were not filtered away. Reads matching the references are on the y-axis. (B and E) and (C and F) shows the mapping of the metagenomes against the K1 and K2 strain, respectively as well as the other reference strains. Ca1 in panes (B and C) and Ca2 in panes (E and F). (G) *B. anthracis* spore counts of soil samples from Ca1 and Ca2 in spores per gram dry soil. Spore counts were estimated by culturing of heat-shocked soil samples and adjusted to dry weight of soil. (EPS 1226 kb)

Additional file 7: Table S3. Comparison of taxonomic classification with four different methods: MetaBIT, Metaxa2, MEGAN, and Kraken. (XLSX 66 kb)

Additional file 8: Figure S4. Comparison of the percentage of classified reads by four metagenomic classification tools. Note the very different scales on the y-axis. Barplots show the relative abundances of classified reads for each time point metagenome of Ca1 (blue) and Ca 2 (orange). The tools are Metaxa2 (A), metaBIT (B), MEGAN (C), Kraken (D). (EPS 1139 kb)

Additional file 9: Table S4. Metaxa2 taxonomic classifications on pair1 16S /18S rRNA sequences. (XLSX 325 kb)

Additional file 10: Figure S5. Soil microbial community relationships by time-point and carcass site. Principal coordinate analysis of a distance matrix created by normalised total counts and using Bray-Curtis dissimilarity. Ca1 is represented by the circles and Ca2 by the triangles, the time-points are visualised in different colours and the arrows are pointing in the direction of increasing days, red arrows for Ca1 and light blue for Ca2. The dotted arrows show the relationship between the day 30 to the Ctrl0 sample. The black arrows indicate the 10 most abundant orders and their effect on the soil microbial communities per time-point. Arrows were fitted using envfit with 1000 permutations using the vegan package in R-studio. (EPS 8 kb)

Additional file 11: Table S5. Overview of the Spearman correlation coefficients of all identified KEGG pathways and their FDR corrected *p*-values. (XLSX 18 kb)

Additional file 12: Table S6. KEGG KO-term abundances for significantly correlating KEGG pathways. Abundances are normalized using DESEQ2 and sorted on carcass 1, time point *T* = 0. (XLSX 368 kb)

Additional file 13: Figure S6. Sample area. (**a**) Ca1, soil samples were taken from within the 30 × 30 cm grid, (**b**) Ca2, samples were taken from within the red square (resembling the 30 × 30 cm metal grid shown in (**a**)). (PNG 749 kb)
