# Supplementary material for: Temporal dynamics in microbial soil communities at anthrax carcass sites
Source: BMC Microbiol. 2017 Sep 26;17:206. doi: 10.1186/s12866-017-1111-6 (PMC5615460; doi:10.1186/s12866-017-1111-6)
Supplement: Supplementary file 1 — Soil nutrients and soil composition at carcass sites. (DOCX 44 kb) [file 12866_2017_1111_MOESM1_ESM.docx]

Supplementary Table S1: Soil nutrients and soil composition at carcass sites.

|  | **Carcass 1** | **Carcass 2** |
| --- | --- | --- |
|  |  |  |
| pH | 8.74 | 8.67 |
| ECw (uS/cm) | 242 | 187 |
| OM (%) | 1.80 | 1.99 |
| N (mg N/kg) | 1264 | 838 |
| P (ppm) | 4.1 | 4.2 |
| K (ppm) | 932 | 1604 |
| Ca (ppm) | 3187 | 3017 |
| Mg (ppm) | 268 | 346 |
| Na (ppm) | 73 | 128 |
| Texture | Sandy loam (55.5 % sand, 34.2 % silt, 10.3 % clay) | Sandy Loam (53.3 % sand, 35.2 % silt, 11.6 % clay) |
