# Supplementary material for: Temporal dynamics in microbial soil communities at anthrax carcass sites
Source: BMC Microbiol. 2017 Sep 26;17:206. doi: 10.1186/s12866-017-1111-6 (PMC5615460; doi:10.1186/s12866-017-1111-6)
Supplement: Supplementary file 4 — A text document contained supplementary methods, equations, results and figure (mentioned only in the supplementary information). (DOCX 896 kb) [file 12866_2017_1111_MOESM4_ESM.docx]

# Supplementary information

## Supplementary methods

*Study area and sample collection*

ENP is a semi-arid savannah with seasonal rainfalls, typically from November through April and a mean annual rainfall in central ENP (Okaukuejo station) of 387 mm (1933-2015). The temperature ranges from an average low in June/July of 6 °C to an average high in October-November of 35 °C (Turner et al., 2013). Anthrax is endemic in ENP and is considered to be a natural part of the ecosystem. Thus the disease is unmanaged, with exception of removing carcasses from waterholes, due to the risk of water contamination. Grazing herbivores such as plains zebra (*Equus quagga*), blue wildebeest (*Connochaetes taurinus*) and springbok (*Antidorcas marsupialis*) are the most common anthrax mortalities (Beyer et al., 2012).

Two fresh zebra anthrax carcasses (hereafter called Ca1 and Ca2) were found on 03.03.2014 between Sprokieswound and Charl Marais Dam, 835 m apart at coordinates S19.031/E015.548 (Ca1) and S19.037/E015.553 (Ca2), respectively. Ca1 was unopened by scavengers, and exhibited as classical anthrax mortality with blood haemorrhaging from the nose, mouth and anus into the soil. In addition, bloating of the carcass caused blood to be expelled through micro-fissures in the skin. Ca2 was minimally scavenged when sampled (through the groin by scavenging vultures), which resulted in less blood leakage from the nose and mouth. Hence, for this carcass the jugular vein was cut to allow blood to seep into the soil. Sampling took place using personal protective equipement (gloves, respirators and eye protection), and we approached the carcass from and remained upwind from it at all times. The sample area was defined as a 30 x 30 cm grid around the focal point of the blood-spill. This was around the nose area of Ca1 (Supplementary information figure 5A (found at the bottom of this document) and the jugular vein area within the red square of Ca2 (Supplementary information figure 5B). Samples were collected into 50 ml tubes from the top 1 to 1.5 cm layer of soil from each. A clean autoclaved spoon was used to fill each tube with soil randomly collected from the sample area, focusing on blood-permeated soil from each time-point to ensure as much consistency as possible within the sampled microbial community. This became more difficult following the first time-point as the area of blood-spill could not be easily visualised, however for the later time-points (day 7 and onwards), random sampling should reduced the heterogeneity among the samples. The tubes were kept in a sealed plastic bag during transport from the sampling sites to the anthrax laboratory at Okaukuejo. Used spoons were kept in a separate sealed bag and washed with 10 % chloride solution and autoclaved upon return to Okaukuejo. Carcass sites were left unaltered to be scavenged and decay naturally. Soil samples were frozen after collection and stored at - 20 °C in a separate freezer for infectious materials until DNA was extracted. Daily rainfall data was measured during the 30-day period at Okaukuejo, located about 40 km from the carcass sites.

*Soil core samples*

For soil analysis of pH and other chemical properties an additional four *B. anthracis* negative soil core samples (10 cm deep) were taken at day 3, at 90° intervals along a 10 m radius from the centre of each carcass. Soil core samples were mixed and analysed for pH, electrical conductivity, organic matter (OM), phosphorous (P), potassium (K), calcium (Ca), magnesium (Mg) and sodium (Na), as well as soil composition of sand, silt and clay were analysed at the Agricultural Laboratory in Windhoek, Namibia. Nitrogen (N) contents were determined using the Kjeldahl method (Horowitz and Chemists, 2002) at Analytical Laboratory Services in Windhoek, Namibia.

*DNA extraction*

DNA was isolated from 250-300mg soli using the FastDNA spin kit for soil, MP Biomedicals with following modifications to the manufacturers protocol. The soil was added to Lysing matrix E tubes, tubes were homogenised with a MiniBead Beater, model 607 (BioSpec Products, Bartlesville, Oklahoma, USA) for 80 sec at 3450 oscillations/min, before being centrifuged at 14 000 revolutions per minute (rpm) (this speed is used for all the centrifugation steps unless otherwise described) for 10 min. After the protein precipitation step, the supernatant is transfered to a 15 ml tube and mixed with the Binding Matrix solution. The solution was mixed on a roter for 2 minutes and then placed in a rack for three minutes, to allow the Binding matrix to settle. 650 μl of the supernatant was removed. The remaining binding matrix solution was then transferred to a SPIN^TM^ Filter and centrifuged for 1 min. The pellet was then resuspended using 500 μl SEWS-M solution, and centrifuged for 3 min without emptying the catch tube. Next, the SPIN^TM^ Filter was transferred to a clean catch tube an allowed to dry for 5 minutes at room temperature. DES buffer was heated to 55 °C in Dri-Bath, type 17600 (Thermolyn, Dubuque, Iowa, USA) before addition to the pellet in the SPIN^TM^ Filter. Then tubes were put on a heat block for 5 min at 55 °C and subsequently centrifuged. Finally the DNA extracts were filter sterilised using an Ultrafree® Durapore PVDF 0.1 µM spinfilter (Millipore, Darmstadt, Germany) by centrifugation at 11.000 (rpm) for 3 min to avoid live cells or spores in the DNA isolate. All non-electrical equipment was autoclaved at 125 °C for 45 min and cleaned with 10 % chloride solution before and after use. All work with the soil samples and extraction of DNA was performed in a Bioflow laminar flow cabinet, tubes were wiped with 10 % chloride solution before and after being taken out of the bench to be homogenised and centrifuged. The tubes containing filtrated DNA was again wiped with 10 % chloride solution before being packed into a cardboard box sealed within a plastic bag in preparation for transport, then the plastic bag was treated with the 10 % chloride solution. All waste was autoclaved at 125 °C for 45 min before incineration.

*Ethanol precipitation*

Ethanol precipitation was performed to safely transport the DNA from ENP to Norway as a pellet. To each sample, 3M CH_2_COONa pH 5.2 in the ratio 1/10 of the sample volume and 2.5 X the sample volume of 100 % ethanol was added, tubes were mixed by inverting the tube 20 times. The samples were then placed on ice for 5 min and centrifuged at 13000 rpm for 40 min. After centrifugation, the supernatant was removed from the samples and pellets were air-dried in a Bioflow laminar flow cabinet for 15 min, tubes were wiped with 10 % chloride solution. After transport, an equal volume of DES buffer, as was removed during the ethanol precipitation, was added to the samples (now considered to be free from live *B. anthracis* spores due to sterile filtration and EtOH precipitation) to ensure no loss of DNA due to changes in volume.

*Qualitative and quantitative analysis of DNA*

Following isolation and purification, the Quant-IT^TM^ PicoGreen® dsDNA Assay kit (Invitrogen, Eugene, Oregon, USA) was used by following the manufacturers protocol and analysed using a FLUOstar® OPTIMA microplate reader (BMG Labtechnologies, Offenburg, Germany) to quantify the DNA. DNA quality was determined with the use of standard NanoDrop analysis and Agilent 2100 Bioanalyser, model G2938C, and the Agilent DNA 7500 Kit (Agilent Technologies, Waldbronn, Germany) following the protocol described by the manufacturer.

*Metagenome sequencing and quality control*

Environmental DNA samples were sequenced at the Norwegian Sequencing Centre (NSC) using the Illumina MiSeq platform (Illumina Inc., San Diego, California, USA). A paired-end sequencing library was generated with a 400 bp insert length and 250 bp reads using a Regular TruSeq adapter ligation kit (Illumina Inc., San Diego, California, USA). Fastq files containing paired-end sequence data were processed using cut-adapt (v1.8) to remove sequence adapters with the following settings: -q: 20; minimum-length: 50; max-n : 1 (Martin, 2011). Next, exact duplicate sequences and low-complexity reads were removed from each dataset using prinseq-lite (v0.20.4) with the following settings: min_qual_mean: 20; trim_qual_left: 20; trim_qual_right: 20; ns_max_n: 1; min_len 50; derep: 1,4; derep_min: 2. For low complexity read removal we used the entropy method with a minimum threshold of 70 (Schmieder and Edwards, 2011).

Average genome size was estimated using MicrobeCensus on the clean paired-end files (Nayfach and Pollard, 2015) with default settings, except for: -n 100000000 -t 32.

*Taxonomic and functional classification of metagenomic reads*

Taxonomy of the metagenomes was determined with four different tools: metaxa2 (v2.0.1) (Bengtsson‐Palme et al., 2015), MEGAN (v5.10.15) (Huson et al., 2007a) , Kraken (0.10.5-beta) (Wood and Salzberg, 2014) and metaBIT (Louvel et al., 2016) Below we describe the procedures followed for each tool to determine the taxonomic composition. Metaxa2 extracts the sequence reads matching the 16S/18S rRNA small subunit (SSU). The quality filter was set to 20, For all further analysis of the metaxa2 data only the pair 1 reads were used, as the taxonomy of pair 1 and pair 2 was close to identical. The unknown section (present in all the taxonomical levels) was removed except for kingdom level to avoid bias of the datasets.

For MEGAN, shotgun sequences from each dataset were compared to the NCBI nr database (accessed on 20.01.2016) using Diamond (v0.7.11) (Buchfink et al., 2015) with settings: blastx --sensitive --max-target-seqs 25 --evalue 0.01 -p 32. The Diamond output was used for taxonomic and functional classification in MEGAN (v5.10.15) with the LCA settings: Min score = 50, Max expected = 0.001, Top percent = 10.0, Min support percent = off, Min support = 5, LCA percent = 100.0, Min complexity = 0.0. (Huson et al., 2007b). Functional classification was determined using KEGG KO-terms and pathways (described below).

Building the Kraken database with the original scripts of the program was no longer possible due to NCBI database update that affected the ftp server structure of the refseq genomes database. We therefore downloaded modified scripts from <https://github.com/mw55309/Kraken_db_install_scripts>, to download refseq genome sequences for: Achaea, Bacteria, Fungi, Protozoa and Viruses. Next we used these downloaded genomes to build a database for Kraken, using the kraken-build command in kraken. Kraken was run with 6 threads, with standard settings. The output was parsed with kraken-filter with a threshold of 0 and a taxonomy report was generated for each sample. The output reports were combined with the script: merge_metaphlan_tables.py, which is part of metaphlan (Truong et al., 2015).

metaBIT was installed following the instructions provided. Classification with metaBIT was done with the following settings for the metaBIT.py script: bowtie2-max-threads:4; metaphlan-max-threads:4, -jre-option: -Xmx16g; -write-config. A costume made yaml file was created to indicate the samples and their location on our computing cluster. Eukaryotic taxa and viruses were included in the classification.

The output from each classification tool was summarized to identify for each dataset the number of raw reads classified. R-studio (version 0.99.489, running R version 3.3.0) was used to visualise the relative abundances of the reads classified for each tool and each dataset. In addition, we calculate for each sample the taxonomic richness and the alpha diversity based on the order level classifications of each metagenomic classifier. The species richness was calculated as the number of order level taxons with at least one read assigned to it. In order to compare the alpha-diversity for each metagenomic classifier we calculated the Shannon-Weaver index using the diversity function implemented in the Vegan package (2.4-2) with the raw counts for each sample and classification tool.

*Visualisation of metaxa2 classifications*

For all further analysis of the metaxa2 data only the pair 1 reads were used, as the taxonomy of pair 1 and pair 2 was close to identical. The unknown section (present in all the taxonomical levels) was removed from all the datasets but at kingdom level to avoid skewness of the datasets. To further investigate the relationship between the diversity at each carcass site, a heatmap was created with the 50 most abundant organisms at each time-point at the order level within the metagenome datasets. This was enabled by first log5 scaling the metaxa2 data and then using decostand with the total option in the vegan (v2.3-5) package to obtain relative abundance. The heatmaps were created by the ggplot2 (v2.1.0) (Wickham, 2009) package in R-Studio.

To visualise the diversity of the metagenome samples, species richness and rarefaction were determined by processing the metaxa2 extracted SSU reads through the MetaAmp 1.1 (<http://ebg.ucalgary.ca/metamp/>) pipeline with these settings: metaamp.pl -map Mapping.txt -an Ca1_Ca2_p1 -seqtype single -seqformat fasta -minoverlen 50 -maxdiffs 0 -truncqual 3 -trunclen 250 -maxee 0.5 -oligos oligos.txt -pdiffs 0. The mapping file was created as described for single reads on <http://ebg.ucalgary.ca/metaamp/html/help.html>.pipeline to obtain operational taxonomical unit (OTU) abundances. OTU richness and rarefaction curves were visualised using R-studio.

*Visualisation of Temporal Dynamics*

A PCoA plot was generated to visualise the temporal species composition. Abundances at the taxonomic order level were first normalised using the order level abundances from the MEGAN analysis using decostand within the vegan package. Sample distances were calculated using the Bray-Curtis dissimilarity measure (vegan v2.3-5). Cmdscale (vegan) was used to calculate the PCoA and the results were visualised with biplot from the ape package (v3.5).

Changes in the metabolic potential of the community over time were analysed by correlating KEGG pathways abundances with AGS size changes over time. Since AGS size behaved differently for samples from Ca1 and Ca2, we analysed them separately. KEGG pathways abundances from each sample were normalised using DESeq2 (Love et al., 2014). Normalised data was correlated with AGS sizes using corr.test (R statistics v3.3.0) using the Spearman correlation method with a False Discovery Rate (FDR) cutoff at 0.05. Correlating KEGG pathways (positive /negative) having a FDR-value < 0.05 were identified and visualized using the pheatmap package (v1.0.8) in R-studio. Significantly correlating KEGG pathways were checked for KO-terms that were uniquely assigned to pathways or not, by downloading the KEGG KO-terms for each pathway map using the KEGG API via ([http://rest.kegg.jp/link/ko/](http://rest.kegg.jp/link/ko/map00010)MAPID), and checking the presence of each KO-term in other maps as well as identifying if KO-terms were observed in the metagenomes in order to check the abundance of functional genes in the metagenomes from the different time-points (Kanehisa and Goto, 2000; Kanehisa et al., 2016). We focused on KEGG metabolic pathways and correlated abundance changes with the AGS.

*Quantitative PCR*

qPCR using *plcR* taqMAMA (Easterday et al., 2005), a *B. anthracis* specific assay that targets the nonsense mutation in the *plcR* gene, was also used to quantify the amount of *B. anthracis* in each DNA sample used for metagenome sequencing (see below). In order to control for extraction efficiency, two 250 mg soil samples from the area around Ca1, but without blood, were spiked with *B. anthracis* Sterne34F2 equal to 3.4 x 10^6^ spores and DNA isolated as described above. The qPCR was performed as described by (Easterday et al., 2005). To each sample, in triplicate, 9 μl of mastermix were added with the following final concentrations: 1 x PCR MasterMix, 1 mg/ml BSA, 1.2 μM forward primer, 1.2 μM reverse primer, 0.25 μM taqMAMA, 1 x EXO IPC Mix (AB, Alameda, California, USA) and 1 x EXO IPC DNA (AB, Alameda, California, USA) on a LightCycler®96 (Roche, Mannheim, Germany) using the LightCycler®Roche 96 software 1.1. The resulting concentrations for each soil sample were then adjusted to the estimated number of genomes per gram of soil (equation further down).

Bacillus anthracis *culture and spore count*

In order to do spore counts on all the soil samples collected, 5 g (2.5 g of the day 3 sample for the cultures for bacterial isolation) was transferred into a 50 ml tube. 45 ml 0.1 % Na_4_O_7_P_2_ was then added and the mixture vortexed at full speed for 10 min. The tubes were then centrifuged for 2 min at 300 relative centrifugal force (rcf) and the supernatant was poured into a new 50 ml tube, the pellet in the old tube was discarded. The supernatant was centrifuged for 15 min at 3000 rcf. The supernatant was then discarded and the pellet re-suspended in 5 ml of dH_2_O, in order to create the stock solution. Stock solution were then heat-shocked by sitting in a water bath at 62.5 °C for 156 min before serial dilutions of 1 ml stock solution, ranging from 10^0^ to 10^-6^. 100 μl from each dilution were plated onto separate polymyxin-lysozyme-EDTA-thallous acetate (PLET) medium (WHO et al., 2008) BD Difco™ brain heart infusion agar (BD diagnostics, Sparks, Maryland, USA). The plates were then incubated at 37 °C for 4 days.

*Bacillus anthracis* colonies were identified based on their morphology and colonies were counted after both 2 and 4 days. For uncertain colonies, the procedure was to re-grow and administer penicillin and gamma phage to determine were penicillin and gamma phage sensitive (WHO et al., 2008). To get an accurate estimate of colony forming unites (CFU) per gram soil, the dilution series that had counts ranging from 30 to 300 CFU, or the lowest dilution with CFUs were used to estimate the number of CFU/g.

From each carcass one colony from the cultures grown for bacterial isolation was chosen from the 10^3^ dilution and re-grown on PLET for 3 days. The plates were then incubated at 37 °C for another 4 days to acquire enough of the bacterium to do DNA isolation, then colonies were scraped of the PLET agar with a spatula and dispensed in 300 μl phosphate buffered saline (PBS) buffer (stock solution Sigma-Aldrich, Saint Louis, Missouri, USA) before DNA isolation as described above.

*Whole genome sequencing and annotation of Bacillus anthracis isolates from zebra carcasses*

A *B. anthracis* isolate from each carcass was obtained by culture as described above. From each carcass one colony was chosen from the 10^3^ dilution and re-grown as a lawn on PLET for 3 days. These isolates were sent for whole genome sequencing at the NSC using the TruSeq Nano reagents (Illumina Inc., San Diego, California, USA) and sequenced with Illumina MiSeq using paired-ends, insert size of 500 bp and a read length of 300 bp. Adapters were removed from raw reads by AdapterRemoval (v1.5.4) (Lindgreen, 2012) with parameters set to –trimns --maxns 1 --trimqualities --minquality 20 --stats --minalignment 50. The quality of the cleaned files was finally checked with SGA preqc (Simpson, 2014).

The isolate genomes were assembled in CLC workbench v.8 using the singleton, pair1 and pair2 truncated files from the AdapterRemoval 1.5.4. The assembled contigs were examined using the Mauve (v2.3.1) aligner (Darling et al., 2004) with standard settings. In order to visualise the coverage of the contigs, reapr (v1.0.17) (Hunt et al., 2013) were run with the options facheck, smaltmap and pipeline, to generate the bam and sam files needed by IGV (v2.3.40) (Robinson et al., 2011) to visualise the contigs. Reapr also evaluated the CLC assembly. In both Mauve, Reapr and IGV the *B. anthracis* Vollum strain was used as reference. NCBI nucleotide blast (Johnson et al., 2008) at http://blast.ncbi.nlm.nih.gov/Blast.cgi was used to check for contamination using default settings. The assembled genome sequences of *B. anthracis* strains Ca1 and Ca2, hereafter referred to as K1 and K2 were annotated using NCBI PGAP pipeline.

*Phylogenetic analysis of whole genomes*

In order to compare the K1 and K2 *B. anthracis* isolates to a larger collection of already known whole genome sequenced *B. anthracis* strains, 109 of the available (as of 06.10.2015) *B. anthracis* genomes on the NCBI genome browser were concatenated into separate pseudochromosomes by the union command within the emboss package (v6.5.7) (Rice et al., 2000). These strains, 111 in total, were uploaded to PanSeq (https://lfz.corefacility.ca/panseq/analyses/) (Laing et al., 2010) and the analysis run with default settings with these exceptions: percent sequence identity cut-off 95 and core genome threshold 111. The resulting snp.phylip file was then analysed in Geneious 8.1 ([http://www.geneious.com](http://www.geneious.com/), (Kearse et al., 2012)) creating a maximum likelihood tree of the phylogenetic relationship between the *B. anthracis* whole genome strains.

*Mapping of metagenome sequences to K1 and K2*

The *B. anthracis* isolate K1 and K2 genomes from Ca1 and Ca2, respectively (Valseth et al., 2016), Genbank accession nr.: LBBZ00000000 and LBCA00000000, respectively) together with whole genomes from *B. cereus* E33L (Assembly nr: GCA_000011625.1), *B. thuringiensis* HD-771 (GCA_000292455.1) and *Bacillus subtilis* 168 (GCA_000009045.1) strains as reference genomes, were investigated by the use of Burrows-Wheeler aligner (BWA) (v0.7.8) (Li and Durbin, 2010). An index was created using bwtsw in bwa index, before BWA-aln mappings was performed with standard settings, and maximum edit distance in the seed set to -k0, to perform the mapping itself. Paired-end alignments were generated with sampe outputting a sam file from the mapping. Reads with mismatches (-tag XM:0) were removed using the bamtools (version 2.4.1) filter option. Finally, idxstats within the SAMtools (v1.3) (Li et al., 2009) package counted the total number of metagenome reads mapping to the reference sequences in the BWA-aln. Results were visualised in R-Studio.

## Supplementary Equations

Calculation of estimated *B. anthracis* genomes per gram soil

Adjusted genomes per μL extract = $\frac{Concentration mean * dilution factor}{Genome weight}*elution$

Adjusted genomes per gram soil = $\frac{Adjusted genomes per gram \mu L extract}{Total soil weight of sample}$

Calculation of percentage recovery of B. anthracis spiked soil sample

Percentage spore recovery = $\frac{Recovered number of genomes (qPCR)}{Original number of genomes}$* 100

## Supplementary results

*Whole genome sequences K1 and K2*

The K1 draft genome consisted 38 contigs covering of 5 456 561 bp, with a GC content of 35.1 %, 5 837 genes and 5 549 CDS. The K2 draft genome was very similar with 38 contigs covering 5 461 141 bp, GC content of 35.1 %, it has 5 856 genes and 5 549 CDS (Valseth et al., 2016). Extracting SNPs for these two isolated as well as the SNPs from 109 of the *B. anthracis* genomes on the NCBI genome browser with PanSeq and generating a maximum likelihood tree with Geneious 8.1 revealed the K1 and K2 strains to belong to the A.Br.Aust94 branch of the *B. anthracis* phylogenetic tree (Keim et al., 2000; Van Ert et al., 2007; Derzelle et al., 2015).

**Supplementary references**

Bengtsson‐Palme, J., Hartmann, M., Eriksson, K.M., Pal, C., Thorell, K., Larsson, D.G.J., and Nilsson, R.H. (2015) Metaxa2: improved identification and taxonomic classification of small and large subunit rRNA in metagenomic data. *Mol Ecol Resour* **15**: 1403-1414.

Beyer, W., Bellan, S., Eberle, G., Ganz, H.H., Getz, W.M., Haumacher, R. et al. (2012) Distribution and molecular evolution of *Bacillus anthracis* genotypes in Namibia. *PLoS Negl Trop Dis* **6**: e1534.

Buchfink, B., Xie, C., and Huson, D.H. (2015) Fast and sensitive protein alignment using DIAMOND. *Nat Methods* **12**: 59-60.

Darling, A.C., Mau, B., Blattner, F.R., and Perna, N.T. (2004) Mauve: multiple alignment of conserved genomic sequence with rearrangements. *Genome Res* **14**: 1394-1403.

Derzelle, S., Girault, G., Kokotovic, B., and Angen, Ø. (2015) Whole genome-sequencing and phylogenetic analysis of a historical collection of *Bacillus anthracis* strains from danish cattle. *PLoS One* **10**: e0134699.

Easterday, W.R., Van Ert, M.N., Zanecki, S., and Keim, P. (2005) Specific detection of *Bacillus anthracis* using a TaqMan mismatch amplification mutation assay. *Biotechniques* **38**: 731-735.

Horowitz, W., and Chemists, A.O.A. (2002) *Official methods of analysis of the AOAC International*: Gaitersburgh, Md: AOAC International.

Hunt, M., Kikuchi, T., Sanders, M., Newbold, C., Berriman, M., and Otto, T.D. (2013) REAPR: a universal tool for genome assembly evaluation. *Genome Biol* **14**: R47.

Huson, D.H., Auch, A.F., Qi, J., and Schuster, S.C. (2007a) MEGAN analysis of metagenomic data. *Genome Res* **17**: 377-386.

Huson, D.H., Auch, A.F., Qi, J., and Schuster, S.C. (2007b) MEGAN analysis of metagenomic data. *Genome Res* **17**: 377-386.

Johnson, M., Zaretskaya, I., Raytselis, Y., Merezhuk, Y., McGinnis, S., and Madden, T.L. (2008) NCBI BLAST: a better web interface. *Nucleic Acids Res* **36**: W5-W9.

Kanehisa, M., and Goto, S. (2000) KEGG: kyoto encyclopedia of genes and genomes. *Nucleic Acids Res* **28**: 27-30.

Kanehisa, M., Sato, Y., Kawashima, M., Furumichi, M., and Tanabe, M. (2016) KEGG as a reference resource for gene and protein annotation. *Nucleic Acids Res* **44**: D457-D462.

Kearse, M., Moir, R., Wilson, A., Stones-Havas, S., Cheung, M., Sturrock, S. et al. (2012) Geneious Basic: an integrated and extendable desktop software platform for the organization and analysis of sequence data. *Bioinformatics* **28**: 1647-1649.

Keim, P., Price, L.B., Klevytska, A.M., Smith, K.L., Schupp, J.M., Okinaka, R. et al. (2000) Multiple-locus variable-number tandem repeat analysis reveals genetic relationships within *Bacillus anthracis*. *J Bacteriol* **182**: 2928-2936.

Laing, C., Buchanan, C., Taboada, E.N., Zhang, Y., Kropinski, A., Villegas, A. et al. (2010) Pan-genome sequence analysis using Panseq: an online tool for the rapid analysis of core and accessory genomic regions. *BMC Bioinformatics* **11**: 461.

Li, H., and Durbin, R. (2010) Fast and accurate long-read alignment with Burrows–Wheeler transform. *Bioinformatics* **26**: 589-595.

Li, H., Handsaker, B., Wysoker, A., Fennell, T., Ruan, J., Homer, N. et al. (2009) The Sequence Alignment/Map format and SAMtools. *Bioinformatics* **25**: 2078-2079.

Lindgreen, S. (2012) AdapterRemoval: easy cleaning of next-generation sequencing reads. *BMC Res Notes* **5**: 337.

Louvel, G., Der Sarkissian, C., Hanghøj, K., and Orlando, L. (2016) metaBIT, an integrative and automated metagenomic pipeline for analysing microbial profiles from high-throughput sequencing shotgun data. *Mol Ecol Resour* **16**: 1415-1427.

Love, M.I., Huber, W., and Anders, S. (2014) Moderated estimation of fold change and dispersion for RNA-seq data with DESeq2. *Genome Biol* **15**: 550.

Martin, M. (2011) Cutadapt removes adapter sequences from high-throughput sequencing reads. *EMBnet journal* **17**: 10-12.

Nayfach, S., and Pollard, K.S. (2015) Average genome size estimation improves comparative metagenomics and sheds light on the functional ecology of the human microbiome. *Genome Biol* **16**: 51.

Rice, P., Longden, I., and Bleasby, A. (2000) EMBOSS: the european molecular biology open software suite. *Trends Genet* **16**: 276-277.

Robinson, J.T., Thorvaldsdóttir, H., Winckler, W., Guttman, M., Lander, E.S., Getz, G., and Mesirov, J.P. (2011) Integrative genomics viewer. *Nat Biotechnol* **29**: 24-26.

Schmieder, R., and Edwards, R. (2011) Quality control and preprocessing of metagenomic datasets. *Bioinformatics* **27**: 863-864.

Simpson, J.T. (2014) Exploring genome characteristics and sequence quality without a reference. *Bioinformatics*: btu023.

Truong, D.T., Franzosa, E.A., Tickle, T.L., Scholz, M., Weingart, G., Pasolli, E. et al. (2015) MetaPhlAn2 for enhanced metagenomic taxonomic profiling. *Nat Methods* **12**: 902-903.

Turner, W.C., Imologhome, P., Havarua, Z., Kaaya, G.P., Mfune, J.K., Mpofu, I.D., and Getz, W.M. (2013) Soil ingestion, nutrition and the seasonality of anthrax in herbivores of Etosha National Park. *Ecosphere* **4**: 13.

Valseth, K., Nesbø, C.L., Easterday, W.R., Turner, W.C., Olsen, J.S., Stenseth, N.C., and Haverkamp, T.H.A. (2016) Draft genome sequences of two *Bacillus anthracis* strains from Etosha National Park, NamibiaDraft genome sequences of two Bacillus anthracis strains from Etosha National Park, Namibia. *Genome Announc* **4**: e00861-00816.

Van Ert, M.N., Easterday, W.R., Huynh, L.Y., Okinaka, R.T., Hugh-Jones, M.E., Ravel, J. et al. (2007) Global genetic population structure of *Bacillus anthracis*. *PLoS One* **2**: e461.

WHO, OiE, and FOA (2008) *Anthrax in humans and animals*. Geneva: World Health Organization Press.

Wickham, H. (2009) *ggplot2: elegant graphics for data analysis*. New York: Springer Science & Business Media.

Wood, D.E., and Salzberg, S.L. (2014) Kraken: ultrafast metagenomic sequence classification using exact alignments. *Genome Biol* **15**: R46.

**Supplementary information figure**


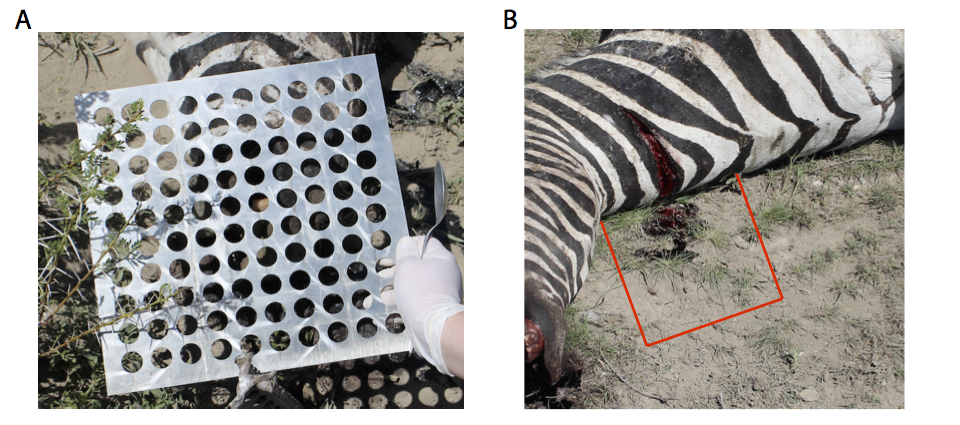


**Supplementary information figure S5:** Sample area. (**a**) Ca1, soil samples were taken from within the 30 x 30 cm grid, (**b**) Ca2, samples were taken from within the red square (resembling the 30 x 30 cm metal grid shown in (**a**)).
