# Supplementary figures and images for: Temporal dynamics in microbial soil communities at anthrax carcass sites
Source: BMC Microbiol. 2017 Sep 26;17:206. doi: 10.1186/s12866-017-1111-6 (PMC5615460; doi:10.1186/s12866-017-1111-6)

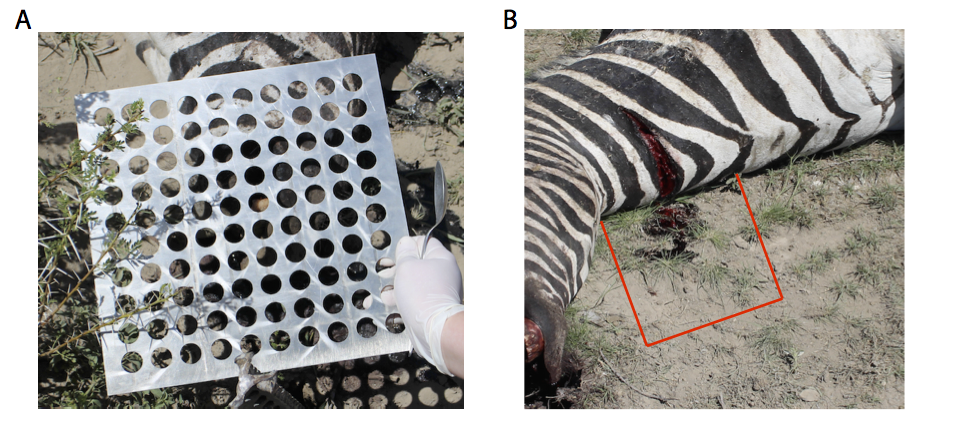

Supplement: Supplementary file 13 — Sample area. (a) Ca1, soil samples were taken from within the 30 × 30 cm grid, (b) Ca2, samples were taken from within the red square (resembling the 30 × 30 cm metal grid shown in (a)). (PNG 749 kb) [file 12866_2017_1111_MOESM13_ESM.png]
